# Lactones 41.† Synthesis and Microbial Hydroxylation of Unsaturated Terpenoid Lactones with *p*-Menthane Ring Systems

**DOI:** 10.3390/molecules18032778

**Published:** 2013-03-01

**Authors:** Aleksandra Grudniewska, Czesław Wawrzeńczyk

**Affiliations:** Department of Chemistry, Faculty of Food Science, Wrocław University of Environmental and Life Sciences, Norwida 25, 50-375 Wrocław, Poland

**Keywords:** lactones, *p*-menthane system, biotransformation, hydroxylation, odoriferous compounds, *Absidia cylindrospora*, *Absidia glauca*, *Syncephalastrum racemosum*

## Abstract

Racemic [(±)-4-isopropyl-1-methyl-7-oxa-*cis*-bicyclo[4.3.0]non-4-en-8-one] and optically active δ,ε-unsaturated lactones [(-)-(1*R*,6*R*)-4-isopropyl-1-methyl-7-oxabicyclo[4.3.0]non-4-en-8-one and (+)-(1*S*,6*S*)-4-isopropyl-1-methyl-7-oxabicyclo[4.3.0]non-4-en-8-one)] with the *p*-menthane system were obtained and their odoriferous properties were evaluated. Biotransformations of the racemic lactone with three fungal strains: *Absidia cylindrospora* AM336, *Absidia glauca* AM177 and *Syncephalastrum racemosum* AM105, were carried out. Microbial transformations afforded hydroxylactones with the hydroxy group in the allylic position.

## 1. Introduction

Lactones with the *p*-menthane system constitute a large group of naturally occurring and synthetic compounds. Many of them, e.g., mintlactone, isomintlactone and wine lactone, are known for their interesting odoriferous properties [1,2,3,4,5,6]. Synthetic lactones of this group possess also valuable biological activity such as antifungal [7] and antifeedant properties [8,9]. 

As a continuation of our interest in the synthesis of odoriferous and biologically active lactones [10], here we present the synthesis of racemic and enantiomeric pairs of new δ,ε-unsaturated lactones with the *p*-menthane system. The odour characteristics of the obtained lactones were also evaluated. The synthesis of enantiomeric lactones was carried out because it is commonly known, that the biological effect of bioactive compounds often depends on their chirality. The complicated stereochemistry-bioactivity relationships of insect pheromones, as an example, has been clearly presented by Kenji Mori [11].

The introduction of the hydroxy group into a molecule often leads to changes in its biological activity. Examples of such dependence are well-documented in the literature, especially among monoterpenes, e.g., limonene and carveol, sabinene and sabinol, α-pinene and verbenol [12,13,14,15]. This information inspired us to check the influence of the hydroxy group in the lactones obtained on their odoriferous properties. 

Encouraged by the literature reports about regio- and stereoselective biohydroxylation of miscellaneous molecules, we applied some fungal strains to this purpose. Filamentous fungal strains *Absidia cylindrospora* [16], *Absidia glauca* [17,18] and *Syncephalastrum racemosum* [19,20] are known for their hydroxylation ability in relation to a broad variety of compounds. The aforementioned fungal strains were applied to transform the racemic δ,ε-unsaturated lactone (±)-**2**.

## 2. Results and Discussion

### 2.1. Synthesis and Odoriferous Properties of Racemic *[(±)-**2**]* and Optically Active *[(–)-(1*R*,6*R*)-**2** and (+)-(1*S*,6*S*)-**2**]*δ,ε-Unsaturated Lactones

**Scheme 1:**
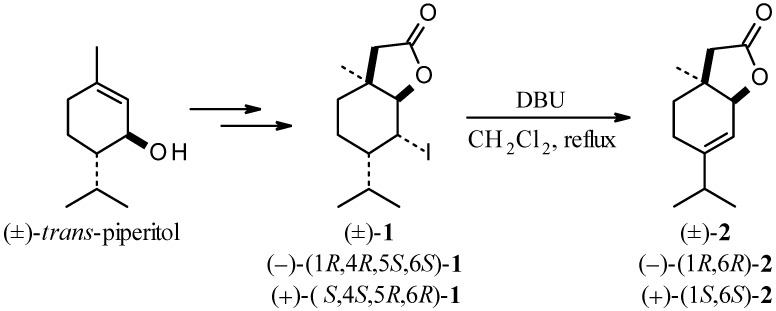
Synthesis of unsaturated lactones (±)-**2**, (–)-**2** and (+)-**2**.

The terpenoid lactone (±)-**2** was obtained in good yield (92%) by dehydrohalogenation of δ-iodo-γ-lactone (±)-**1**, which was synthesized from racemic *trans-*piperitol as described earlier (Scheme 1) [21]. 1,8-Diazabicyclo[5.4.0]undec-7-ene (DBU) was applied as the base in elimination reaction. The structure of lactone (±)-**2** was confirmed by its spectral data. The multiplet at 5.53 ppm of proton H-5 in the ^1^H-NMR spectrum of (±)-**2** confirmed the presence of the double bond. The shape of the of H-6 proton multiplet (δ = 4.42, doublet, *J* = 3.4 Hz) and the absence of signals of the H-4 proton also indicated a C4-C5 double bond. Additionally, in the ^13^C-NMR spectrum of lactone (±)-**2** signals at 114.65 and 150.81 ppm from carbon atoms C4 and C5, respectively, were present.

Racemic δ,ε-unsaturated lactone (±)-**2** possesses a weak odour slightly resembling that of coumarin. Knowing that fragrance of compounds depends on their chirality, we decided to synthesize two optically active unsaturated lactones: (–)-**2** and (+)-**2**. These compounds (–)-(1*R*,6*R*)-**2** and (+)-(1*S*,6*S*)-**2** were obtained from the corresponding iodolactones (–)-(1*R*,4*R*,5*S*,6*S*)-**1** and (+)-(1*S*,4*S*,5*R*,6*R*)-**1**, respectively, similarly to the synthesis of racemic lactone (±)-**2** (Scheme 1). The enantiomeric excesses of (–)-(1*R*,6*R*)-**2** and (+)-(1*S*,6*S*)-**2**, as determined by chiral GC, were 98% and 96%, respectively (the same as the starting iodolactones). The odoriferous properties of the enantiomerically enriched lactones (–)-**2** and (+)-**2** were also evaluated. The lactone (–)-(1*R*,6*R*)-**2** possesses an intense, dill odour, while the fragrance of (+)-(1*S*,6*S*)-**2** is medium intensity and coumarin-like (Table 1). The comparison of odoriferous properties of racemic and enantiomeric lactones confirms the rule that usually an odour of compound strongly depends on the configuration of chiral centres present in the molecule.

**Table 1:** Odoriferous characteristics of lactones synthesized.

Compound	Odour description
(±)- **2**	Weak, slightly similar to (+)- **2**
(–)-(1 *R,*6*R*)-**2**	Intensive, dill
(+)-(1 *S,*6*S*)-**2**	Medium intensity, coumarin-like

### 2.2. Biotransformation of Racemic δ,ε-Unsaturated Lactone *[(±)-**2**]*

**Scheme 2:**
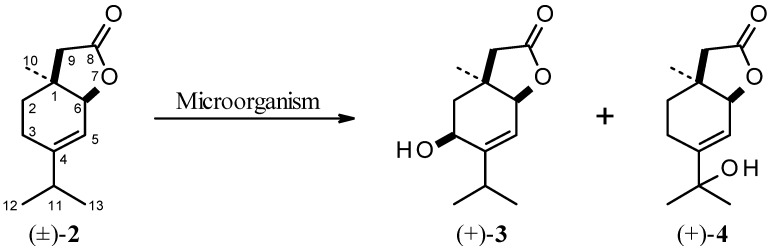
Biotransformation products of lactone (±)-**2**.

Six fungi strains were tested for their ability to transform racemic lactone (±)-**2**. Three of them: *Absidia cylindrospora* AM336, *Absidia glauca* AM177 and *Syncephalastrum racemosum* AM105 transformed the substrate into the same products – hydroxylactones **3** and **4** (Scheme 2). No formation of any biotransformation products of (±)-**2** in the cultures of another three microorganisms (*Fusarium culmorum* AM3/1, *F. oxysporum* AM145 and *Penicillium vinaceum* AM110), was observed after 10 days of incubation.

The progress of biotransformation was monitored by chiral gas chromatography. Hydroxylactone **4** was the major product in all biotransformations carried out. The enantioselectivity of its formation depended on the fungi strains applied. The composition of the products mixture of biotransformation of (±)-**2** by selected microorganisms are presented in Table 2.

**Table 2:** The composition (according to chiral GC) of the products mixture in the course of preparative microbial transformation of lactone (±)-**2**.

Microorganism	Time (days)	2			3				4 *	
		(%)	*ee* (%)	Config.	(%)	*ee* (%)	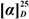 **	Config.	(%)	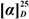 **
*A. cylindrospora* AM336	1	66	*3*	(1*S*,6*S*)	14	*14*		(1*S*,3*S*,6*S*)	20	
	2	41	*0*	-	22	*36*		(1*S*,3*S*,6*S*)	37	
	4	0	-	-	39	*39*	+8.3 (*c* 0.38)	(1*S*,3*S*,6*S*)	61	+2.2 (*c* 0.23)
*A. glauca* AM177	2	67	*7*	(1*S*,6*S*)	6	*0*		-	27	
	4	32	*6*	(1*S*,6*S*)	15	*20*		(1*S*,3*S*,6*S*)	53	
	6	9	*9*	(1*S*,6*S*)	23	*22*	+5.1 (*c* 0.10)	(1*S*,3*S*,6*S*)	68	+0.8 (*c* 1.28)
*S. racemosum* AM105	2	43	*72*	(1*S*,6*S*)	17	*18*		(1*S*,3*S*,6*S*)	40	
	4	11	*54*	(1*S*,6*S*)	20	*35*		(1*S*,3*S*,6*S*)	69	
	6	8	*50*	(1*S*,6*S*)	21	*38*	+6.8 (*c* 0.36)	(1*S*,3*S*,6*S*)	71	+1.1 (*c* 1.19)

* enantiomeric excess was not determined; ** in CHCl_3_.

After the first day of incubation (±)-**2** in the culture of *A*. *cylindrospora* AM336 the product mixture contained 66% of unreacted substrate (**2**), 14% of hydroxylactone **3** and 20% of product **4**. In the next days the amount of **2** decreased and after four days the formation of 39% of **3** and 61% of **4** was observed. The enantiomeric excess of hydroxylactone (+)-**3** obtained in this way was the highest one and reached a value of 39%.

The preparative biotransformation of unsaturated lactone (±)-**2** (120 mg) in the shaken cultures of *A*. *cylindrospora* AM336 after four days afforded: 15 mg (12% isolated yield) of (+)-3-hydroxy-4-isopropyl-1-methyl-7-oxa-*cis*-bicyclo[4.3.0]non-4-en-8-one (**3**) (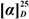 = +8.3° (*c* 0.38, CHCl_3_), *ee* = 39%) and 28 mg (22% isolated yield) of (+)-4-(1'hydroxy-1'methylethyl)-1-methyl-7-oxa-*cis*-bicyclo[4.3.0]non-4-en-8-one (**4**) (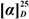 = +2.2° (*c* 0.23, CHCl_3_).

The structure of these products was confirmed by their spectral data. The presence of the hydroxy group in products **3** and **4** was confirmed by absorption bands in the IR spectra at 3438 and 3450 cm^−1^, respectively. Furthermore, the absorption bands at 1760 and 1773 cm^−1^, respectively, indicated that the γ-lactone ring has been retained. The final proof of the structure of hydroxylactones **3** and **4** was delivered by NMR spectroscopy.

Analysis of the ^1^H-NMR spectrum of product **3** indicates that biohydroxylation had taken place at the C-3 position. The presence of multiplet at 4.37 ppm for proton H-3 and the change of multiplicity and chemical shift of protons CH_2_-2 (compared to the spectrum of the substrate) confirm this assignation. The signal from the pseudoequatorial H-2 proton (δ = 2.07) gave a doublet of doublets with a geminal coupling constant *J*_H2a-H2e_ = 14.0 Hz and a small coupling constant *J* = 5.2 Hz with the pseudoequatorial H-3 proton. The pseudoaxial H-2 proton also gave a doublet of doublets (δ = 1.67) with the same geminal coupling constant (*J*_H2a-H2e_ = 14.0 Hz) and with the vicinal coupling constant *J* = 7.1 Hz with the pseudoequatorial H-3 proton. On the basis of these data and according to model analysis (Dreiding models) a pseudoequatorial position was assigned to the H-3 proton. This analysis indicates also that the H-3 proton is situated close to the plane of the double bond, so it is deshielded and its signal is located at 4.37 ppm. Additionally, a significant increase in the chemical shift difference of the CH_2_-9 protons (0.22 ppm) in comparison to difference in the spectrum of (±)-**2** (0.08 ppm) indicates the nearness of the hydroxy group to one of these protons and the *cis*-orientation of the OH group to the γ-lactone ring in product **3**. The fungal strain *A*. *cylindrospora* AM336 is known for its allylic position hydroxylation ability. Our previous biotransformation of unsaturated lactones with *A*. *cylindrospora* AM336 afforded hydroxylactones with pseudoaxially oriented hydroxy groups in the allylic position [22,23].

The next product **4** of the biotransformation of (±)-**2** was also identified as a hydroxylactone with the hydroxy group in the allylic position. Spectral data provided evidence that the hydroxy group was introduced in the C-11 position. The methyl groups at C-11 gave two singlets (δ = 1.34 and 1.35) whereas in the spectrum of substrate (±)-**2** two doublets (*J* = 6.8 Hz) at 1.01 and 1.02 ppm were observed. Disappearance of the signal from H-11 and the lack of new signals between 3–4 ppm confirm the structure of lactone **4**. Additionally, in the ^13^C-NMR spectrum the signal from the carbon atom directly connected to the hydroxy group was situated at 72.61 ppm. Moreover, the absence of this signal in the DEPT135 spectrum undoubtedly confirms of the position of hydroxy group in product **4** at C-11.

*A. glauca* AM177 showed lower activity for the conversion of (±)-**2** than *A*. *cylindrospora* AM336 (Table 2). Six days of incubation afforded a mixture of products containing still 9% of substrate (+)-**2**, 23% of hydroxylactone (+)-**3** and 68% of product (+)-**4**. This biocatalyst produced racemic hydroxylactone (±)-**3** in the second day of incubation, but after six days of biotransformation (+)-**3** isomer predominated (*ee* = 22%) in isolated final product.

The preparative biotransformation of lactone (±)-**2** (120 mg) in the shaken culture of *A. glauca* AM177 after six days gave: 5 mg (4% isolated yield) of (+)-3-hydroxy-4-isopropyl-1-methyl-7-oxa-*cis*-bicyclo[4.3.0]non-4-en-8-one (**3**) (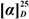 = +5.1 (*c* 0.10, CHCl_3_), *ee* = 22%) and 21 mg (16% isolated yield) of (+)-4-(1'hydroxy-1'methylethyl)-1-methyl-7-oxa-*cis*-bicyclo[4.3.0]non-4-en-8-one (**4**) (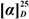 = +0.8° (*c* 1.28, CHCl_3_).

Hydroxylactone **4** was also as the main product of transformation of (±)-**2** in the culture of *S*. *racemosum* AM105. On the second day of biotransformation *S*. *racemosum* AM105 produced (+)-**3** with 18% enantiomeric excess. In the next days the enantioselectivity of this process was increased and finally (+)-isomer of **3** was obtained with 38% enantiomeric excess. Six days of incubation of lactone (±)-**2** (120 mg) afforded: hydroxylactones **3** (7 mg, 5% isolated yield, 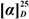 = +6.8 (*c* 0.36, CHCl_3_), *ee* = 38%) and **4** (29 mg, 22% isolated yield, 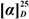 = +1.1° (*c* 1.19, CHCl_3_)).

Additionally, to confirm the absolute configuration of products (+)-**3** and (+)-**4** obtained from the conversion of (±)-**2**, enantiomerically enriched substrate (–)-**2** (*ee* = 98%), with known absolute configuration (1*R*,6*R*) was subjected to the biotransformation. Microbial transformation of (–)-(1*R*,6*R*)-**2** was performed on analytical scale by *A*. *cylindrospora* AM336.

The retention time of product **3** obtained from the conversion of (–)-(1*R*,6*R*)-**2** was *t*R = 115.45 min and the retention times of the two enantiomers of **3** formed during the biotransformation of (±)-**2** were *t*R = 114.30 and 115.45 min, respectively. These results indicate that stereoisomer with *t*R = 115.45 min has *R* configuration at C-1 and C-6. Knowing that the hydroxy group at C-3 is *cis*-oriented with respect to the γ-lactone ring it was possible to ascribe the absolute configuration at this chirality centre. On the basis of these data 1*S*,3*S*,6*S* configuration of (+)-isomer of **3** with *t*R = 114.30 min and 1*R*,3*R*,6*R* configuration of the (–)-enantiomer of **3** with *t*R = 115.45 min were assigned.

Unfortunately, it was impossible to evaluate the enantiomeric excess of hydroxylactone **4** by chromatographic methods using the chiral columns at our disposal. However, the analysis of the composition of the products mixture obtained from transformation of lactone (±)-**2** (Table 2) indicates that 1*R*,6*R* isomer of **2** was transformed faster than the 1*S*,6*S* one, so it could be suggested that the (+)-isomer of **4** has a 1*R*,6*R* configuration.

## 3. Experimental

### 3.1. General

Unless otherwise stated, all chemicals were purchased as the highest purity commercially available and were used without further purification. DBU (1,8-diazabicyclo[5.4.0]undec-7-ene) was purchased from Fluka (Poznań, Poland). ^1^H-NMR, ^13^C-NMR, DEPT 135 (distortionless enhancement by polarisation transfer), ^1^H–^1^H COSY (correlation spectroscopy) and HSQC (heteronuclear single quantum correlation) spectra were recorded in CDCl_3_ solution on a Bruker Avance^™^ 600 (600 MHz) spectrometer. Chemical shifts were referenced to the residual solvent signal (δ_H_ = 7.26, δ_C_ = 77.00). IR spectra were recorded for liquid films on a Thermo-Nicolet IR300 FT-IR spectrometer. HRCIMS was measured on a JEOL JMS-700 MS station. CH elemental analyses were performed on a CE Instruments EA-1110 elemental analyzer. Optical rotations were determined on an Autopol IV automatic polarimeter (Rudolph) in chloroform solutions, concentrations denoted in g/100 mL. Analytical TLC was performed on Fluka Kieselgel 60, F_254_ plates with the solvent system hexane/diethyl ether, 4:1. Compounds were detected by spraying the plates with 1% Ce(SO_4_)_2_, 2% H_3_[P(Mo_3_O_10_)_4_] in 10% H_2_SO_4_, followed by heating to 120 °C. Column chromatography was performed on silica gel (Kieselgel 60, 230–400 mesh ASTM, Merck) with a mixture of hexane and diethyl ether (in various ratios) as eluent. Gas chromatography analysis was carried out on an Agilent 6890N GC (FID, carrier gas H_2_). Enantiomeric excesses were determined on a CP-Chirasil-DEX CB column (25 m × 0.25 mm × 0.25 μm) with the following temp. Program: 90 °C, 155 °C (0.5 °C/min), 200 °C (20 °C/min) (10 min). The total run time was 142.25 min; *t*_R_ (–)-(1*R*,6*R*)-**2** = 58.04 min, *t*_R_ (+)-(1*S*,6*S*)-**2** = 58.72 min, *t*_R_** 4** = 109.37 min, *t*_R_ (+)-(1*S*,3*S*,6*S*)-**3** = 114.30 min, *t*_R_ (–)-(1*R*,3*R*,6*R*)-**3** = 115.45 min.

### 3.2. Chemical Synthesis

Lactones (±)-**2**, (–)-**2** and (+)-**2** were synthesized from corresponding δ-iodo-γ-lactones (±)-**1**, (–)-**1** and (+)-**1**, respectively, which were obtained starting from corresponding *trans*-piperitols as described earlier [21]. Dehydrohalogenation of iodolactones was carried out according to the following procedure: to the solution of δ-iodo-γ-lactones (3 mmol) in methylene chloride (20 mL), 1,8-diazabicyclo[5.4.0]undec-7-ene (DBU, 6 mmol) was added and the mixture was refluxed for 2 h. After evaporating the solvent, the residue was diluted with diethyl ether and washed with saturated solution of NH_4_Cl. The ethereal extract was washed with brine, dried over anhydrous MgSO_4_ and concentrated *in vacuo*. The crude product was purified by column chromatography (hexane/diethyl ether, 2:1). The yields of the reactions, physical and spectral data of the unsaturated lactones obtained are given below:

(±)*-4-Isopropyl-1-methyl-7-oxa-cis-bicyclo[4.3.0]non-4-en-8-one* [(±)**-2**]. Lactone (±)-**2** (yellow oily liquid, 0.84 g, yield 92%) was obtained from (±)-5-iodo-4-isopropyl-1-methyl-7-oxa-*cis*-bicyclo[4.3.0]nonan-8-one [(±)-**1**, 1.5 g, 4.46 mmol]; ^1^H-NMR (CDCl_3_) δ: 1.01 and 1.02 (two d, *J* = 6.8 Hz, 6H, (C*H*_3_)_2_CH-), 1.13 (s, 3H, CH_3_-1), 1.53 (ddd, *J* = 13.5, 10.7 and 5.2 Hz, 1H, one of CH_2_-2), 1.62 (ddd, *J* = 13.5, 6.8 and 6.8 Hz, 1H, one of CH_2_-2), 2.05 (m like t, *J* = 5.9 Hz, 2H, CH_2_-3), 2.26 (septet, *J* = 6.8 Hz, 1H, (CH_3_)_2_C*H*-), 2.33 and 2.41 (two d, *J* = 17.0 Hz, 2H, CH_2_-9), 4.42 (d, *J* = 3.4 Hz, 1H, H-6), 5.53 (m, 1H, H-5); ^13^C-NMR (CDCl_3_) δ: 20.83 and 21.11 ((*C*H_3_)_2_CH–), 22.77 (C-3), 22.94 (CH_3_-1), 30.03 (C-2), 34.94 ((CH_3_)_2_*C*H-), 36.83 (C-1), 42.30 (C-9), 82.34 (C-6), 114.65 (C-5), 150.81 (C-4), 176.28 (C-8); IR (film, cm^−1^): 2962 (m), 1778 (s), 1664 (w), 1384 (w), 1364 (w), 1157 (m); elemental analysis calcd (%) for C_12_H_18_O_2_ (194.28): C 74.19, H 9.34; found: C 73.51, H 9.59.

(-)*-(1R,6R)-4-Isopropyl-1-methyl-7-oxabicyclo[4.3.0]non-4-en-8-one* [(-)-**2**]. Lactone (-)-**2** (yellow oily liquid, 0.27 g, yield 91%, *ee* = 98%) was obtained from (–)-(1*R*,4*R*,5*S*,6*S*)-5-iodo-4-isopropyl-1-methyl-7-oxabicyclo[4.3.0]nonan-8-one ((-)-**1**) (0.5 g, 1.55 mmol, *ee* = 98%). 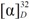 = −1.9° (*c* 1.15, CHCl_3_). IR and NMR spectra were identical with those of (±)-**2**.

(+)*-(1S,6S)-4-Isopropyl-1-methyl-7-oxabicyclo[4.3.0]non-4-en-8-one* [(+)-**2**]. Lactone (+)-**2** (yellow oily liquid, 0.28 g, yield 94%, *ee* = 96%) was obtained from (+)-(1*S*,4*S*,5*R*,6*R*)-5-iodo-4-isopropyl-1-methyl-7oxabicyclo[4.3.0]nonan-8-one ((+)-**1**) (0.5 g, 1.55 mmol, *ee* = 96%). 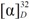 = +2.3° (*c* 1.24, CHCl_3_). IR and NMR spectra were identical with those of (±)-**2**.

### 3.3. Microorganisms

The following fungal strains were used for screening: *Absidia cylindrospora* AM336, *Absidia glauca* AM177, *Fusarium culmorum* AM3/1, *Fusarium oxysporum* AM145, *Penicillium vinaceum* AM110 and *Syncephalastrum racemosum* AM105. The microorganisms came from the Collection of the Institute of Biology and Botany Medical University, Wrocław. They were maintained at 4 °C on Sabouraud agar slants containing peptone (10 g), glucose (40 g) and agar (15 g) dissolved in water (1 L) at pH 5.7.

### 3.4. Screening-Scale Biotransformations

Screening scale biotransformations of lactone (±)-**2** by six microorganisms were carried out in 300 mL Erlenmeyer flasks. The corresponding microorganisms were cultivated at room temperature in Erlenmeyer flasks containing 100 mL medium, which had the following composition (per 1 L of distilled water): peptone (10 g) and glucose (30 g). Cultures were incubated in a rotary shaker at 150 rpm at 25 °C. After 3–5 days, 10 mg of substrate (±)-**2** dissolved in 1 mL of acetone was added to the shaken cultures. The samples of biotransformation mixture were extracted with chloroform after 1, 2, 4 and 6 days. They were dried over anhydrous MgSO_4_, concentrated *in vacuo* and analyzed by TLC and GC.

The same procedure was applied during the transformation of (–)-**2** by *Absidia cylindrospora* AM336.

Additionally, two control flask were used for each biotransformations. A culture control contained sterile culture medium with microorganism inoculum. This experiment used to define and exclude the secondary metabolites generated by fungi. The second control flask contained substrate and sterile growth medium incubated without fungi. The substrates used for the biotransformation were stable in these conditions. It can be concluded that all products isolated from the cultures were bioconversion products.

### 3.5. Preparative-Scale Biotransformations

Preparative scale biotranasformations were carried out in 2 L flat bottomed flasks containing 400 mL of medium (the same as in the screening scale). Lactone (±)-**2** (120 mg in 1 mL acetone) was added to the grown cultures of corresponding microorganisms (*Absidia cylindrospora* AM336, *Absidia glauca* AM177, *Syncephalastrum racemosum* AM105) prepared as described in the screening procedure. After 4 or 6 days the products were extracted with chloroform (3 × 150 mL). The organic solutions were dried over anhydrous MgSO4 and concentrated *in vacuo*. Mixtures of products as well as metabolites produced by the fungi were separated by column chromatography (hexane-diethyl ether, 9:1 → 4:1). The spectral data of biotransformation products are given below:

*3-Hydroxy-4-isopropyl-1-methyl-7-oxa-cis-bicyclo[4.3.0]non-4-en-8-one* (**3**). Colourless oily liquid. ^1^H-NMR (CDCl_3_) δ: 1.07 and 1.10 (two d, *J* = 6.9 Hz, 6H, (C*H*_3_)_2_CH–), 1.29 (s, 3H, CH_3_-1), 1.62 (s, 1H, –OH), 1.67 (dd, *J* = 14.0 and 7.1 Hz, 1H, H-2, pseudoaxial), 2.07 (dd, *J* = 14.0 and 5.2 Hz, 1H, H-2, pseudoequatorial), 2.22 and 2.44 (two d, *J* = 17.1 Hz, 2H, CH_2_-9), 2.63 (septet, *J* = 6.9 Hz, 1H, CH_3_)_2_C*H*-), 4.37 (m, 1H, H-3), 4.60 (m, 1H, H-6), 5.55 (m, 1H, H-5); ^13^C-NMR (CDCl_3_) δ: 20.93 and 22.23 ((*C*H_3_)_2_CH–), 26.39 (CH_3_-1), 29.72 ((CH_3_)_2_*C*H-), 37.52 (C-1), 39.91 (C-2), 40.70 (C-9), 64.95 (C-3), 82.46 (C-6), 117.59 (C-5), 150.98 (C-4), 176.02 (C-8); IR (film, cm^−1^): 3438 (b, s), 2961 (s), 1760 (s), 1168 (m), 1014 (m), 956 (m). HRCIMS [M+H]^+^* m/z* 211.1333 (calcd for C_12_H_19_O_3_, 211.1332).

*4-(1'Hydroxy-1'methylethyl)-1-methyl-7-oxa-cis-bicyclo[4.3.0]non-4-en-8-one* (**4**). Colourless oily liquid. ^1^H-NMR (CDCl_3_) δ: 1.15 (s, 3H, CH_3_-1), 1.34 and 1.35 (two s, 6H, (C*H*_3_)_2_C(OH)–), 1.52 (s, 1H, –OH), 1.58 (dt, *J* = 13.6 and 5.4 Hz, 1H, one of CH_2_-2), 1.66 (m, 1H, one of CH_2_-2), 2.13 (m, 1H, one of CH_2_-3), 2.21 (m like dt, *J* = 18.1 and 5.3 Hz, 1H, one of CH_2_-3) 2.36 and 2.43 (two d, *J* = 17.1 Hz, 2H, CH_2_-9), 4.48 (d, *J* = 4.1 Hz, 1H, H-6), 5.89 (m, 1H, H-5); ^13^C-NMR (CDCl_3_) δ: 21.10 (C-3), 22.99 (CH_3_-1), 28.64 and 28.84 ((*C*H_3_)_2_C(OH)–), 30.21 (C-2), 36.57 (C-1), 42.12 (C-9), 72.61 ((CH_3_)_2_*C*(OH)–), 81.96 (C-6), 114.82 (C-5), 150.84 (C-4), 176.09 (C-8); IR (film, cm^−1^): 3450 (b, s), 2972 (s), 1773 (s), 1199 (s), 956 (s). HRCIMS [M+H]^+^* m/z* 211.1328 (calcd for C_12_H_19_O_3_, 211.1322).

## 4. Conclusions

Racemic (±)-**2** and an enantiomeric pair of unsaturated γ-lactones with the *p*-menthane system (–)-**2** and (+)-**2** were synthesized and their odoriferous properties were evaluated. Comparison of the odour of racemic (±)-**2** and optically active compounds (–)-**2** and (+)-**2** confirmed that their fragrance depends on the configuration of their chiral centers.

The results obtained in this work indicate that hydroxylation of (±)-**2** by all fungi strains studied has taken place in the allylic positions C-3 and C-11. Similar to the literature data [24] the product with the hydroxy group at C-11 was formed preferentially. Probably it is the result of easier oxidation of tertiary C–H bond than the primary or secondary ones. Unfortunately, in this case, the introduction of the hydroxy group into the substrate molecule resulted in a loss of odoriferous properties. 

## Supplementary Materials

Supplementary materials can be accessed at: http://www.mdpi.com/1420-3049/18/3/2778/s1.
