# Supplementary material for: Lactones 41. Synthesis and Microbial Hydroxylation of Unsaturated Terpenoid Lactones with p-Menthane Ring Systems
Source: Molecules. 2013 Mar 1;18(3):2778–87. doi: 10.3390/molecules18032778 (PMC6269982; doi:10.3390/molecules18032778)

Supplementary Materials

List of contents

^{1}H-NMR spectrum of **2** (Figure 1)…………………………………………………………...S1

^{13}C-NMR spectrum of **2** (Figure 2)…………………………………………………............. S2

^{1}H-NMR spectrum of **3** (Figure 3)……………………………………………….…………..S2

^{13}C-NMR spectrum of **3** (Figure 4)…………………………..………………………........S3

^{1}H-NMR spectrum of **4** (Figure 5)…………………………………………………………...S3

^{13}C-NMR spectrum of **4** (Figure 6)……………………………..…………………………....S4

CGC chromatograms of lactones (±)-**2**, (+)-**2** and (–)-**2** (Figure 7)..……………………….. S4

Figure 1. ^{1}H-NMR ($CDCl_3$, 600 MHz) spectrum of **2**.

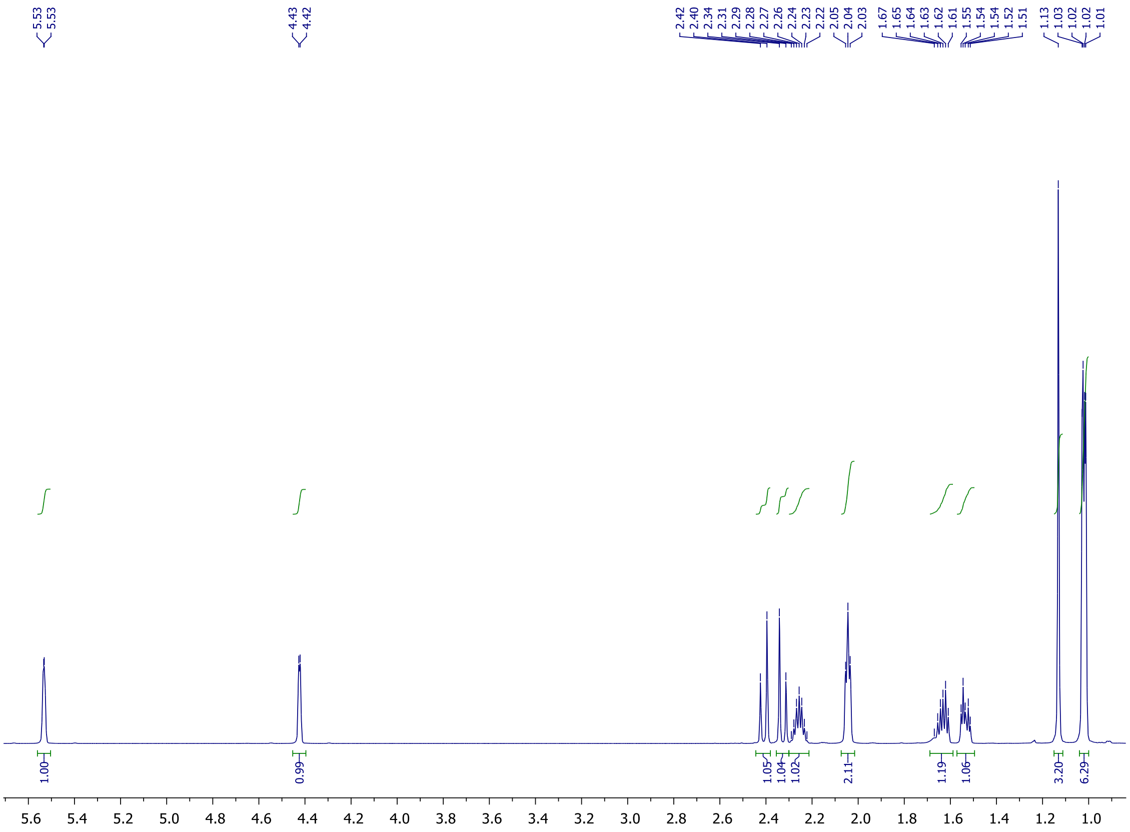

Figure 2. ^{13}C-NMR ($CDCl_3$, 151 MHz) spectrum of **2**.

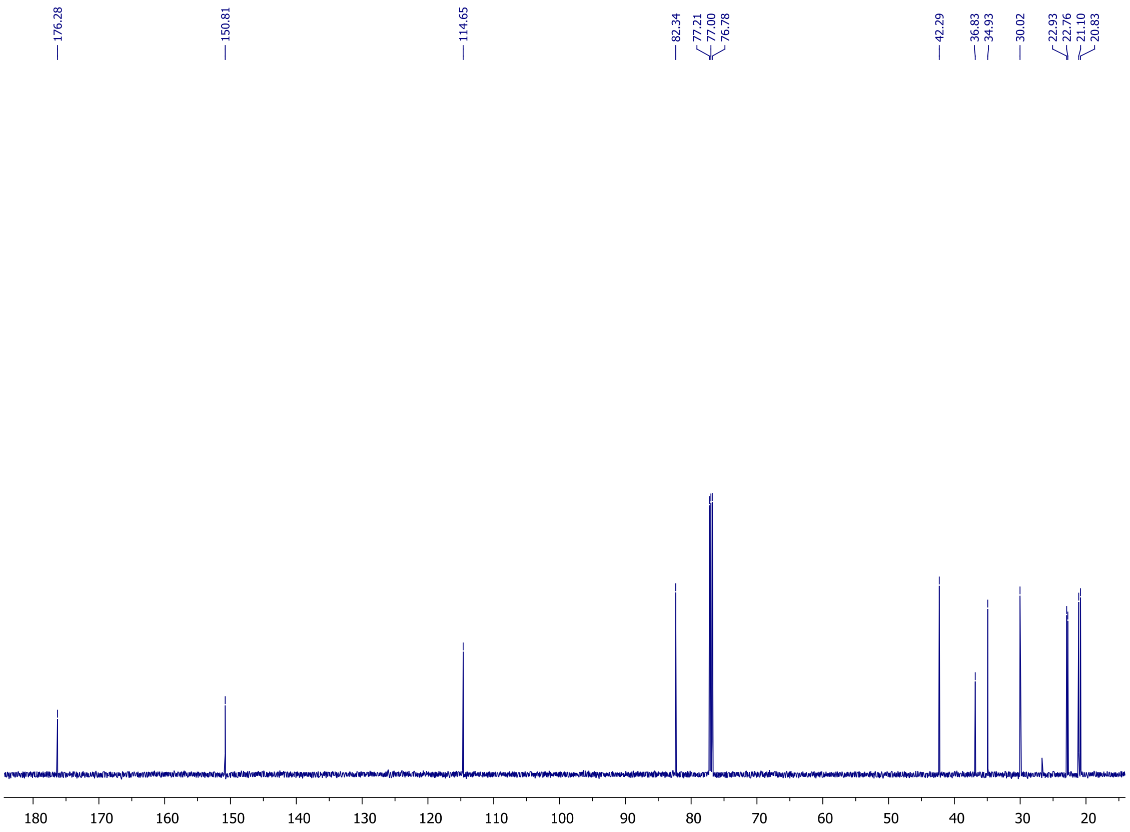

Figure 3. ^{1}H-NMR ($CDCl_3$, 600 MHz) spectrum of **3**.

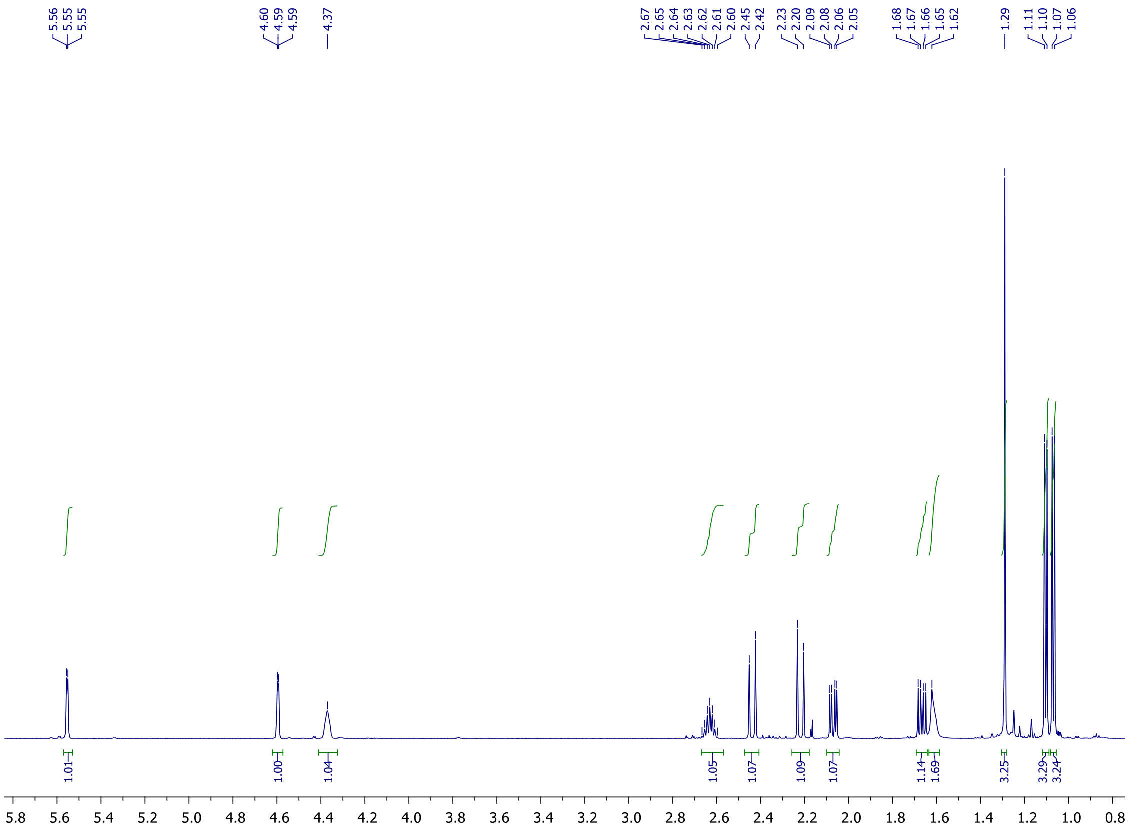

Figure 4. ^{13}C-NMR ($CDCl_3$, 151 MHz) spectrum of **3**.

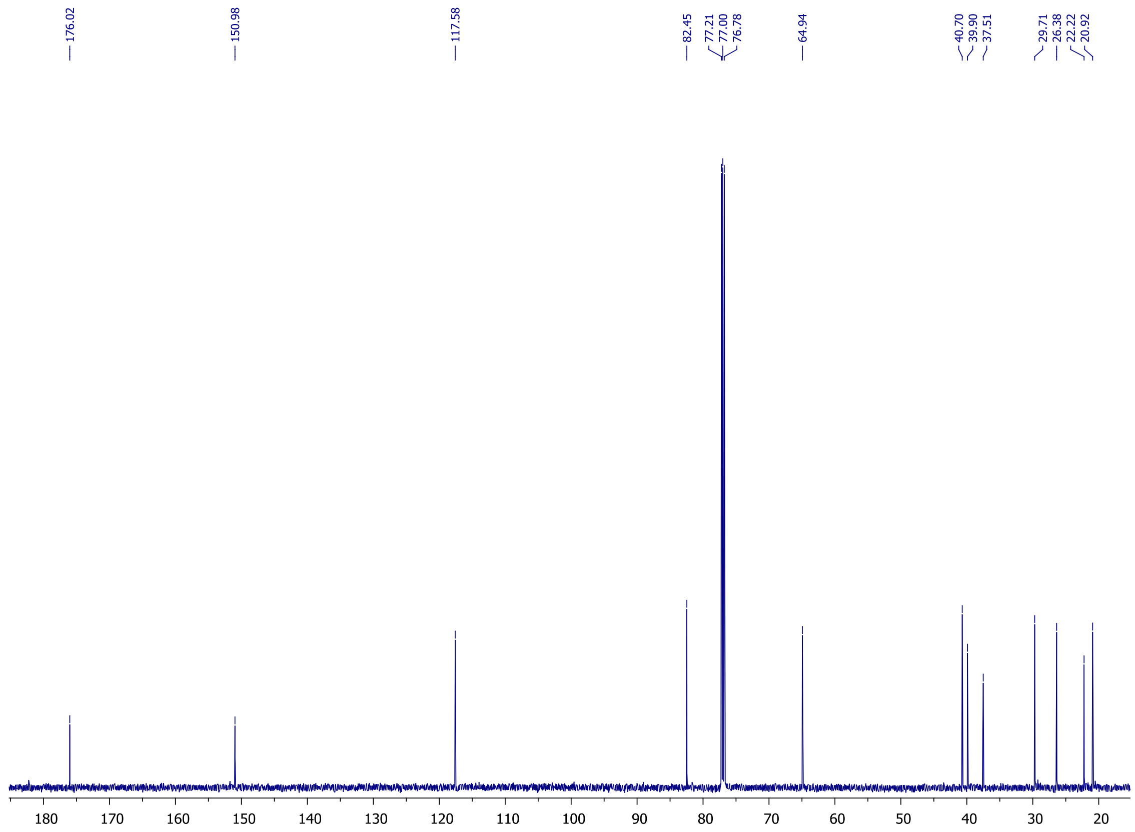

Figure 5. ^{1}H-NMR ($CDCl_3$, 600 MHz) spectrum of **4**.

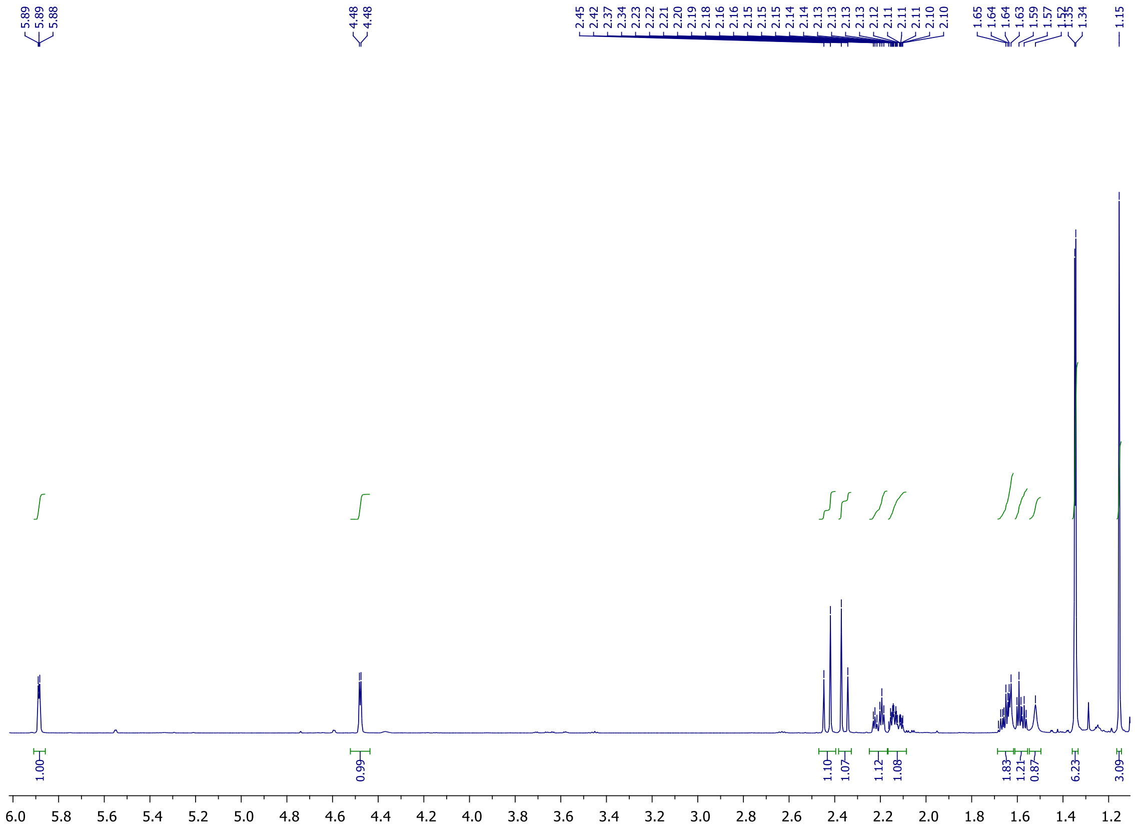

Figure 6. ^{13}C-NMR ($CDCl_3$, 151 MHz) spectrum of **4**.

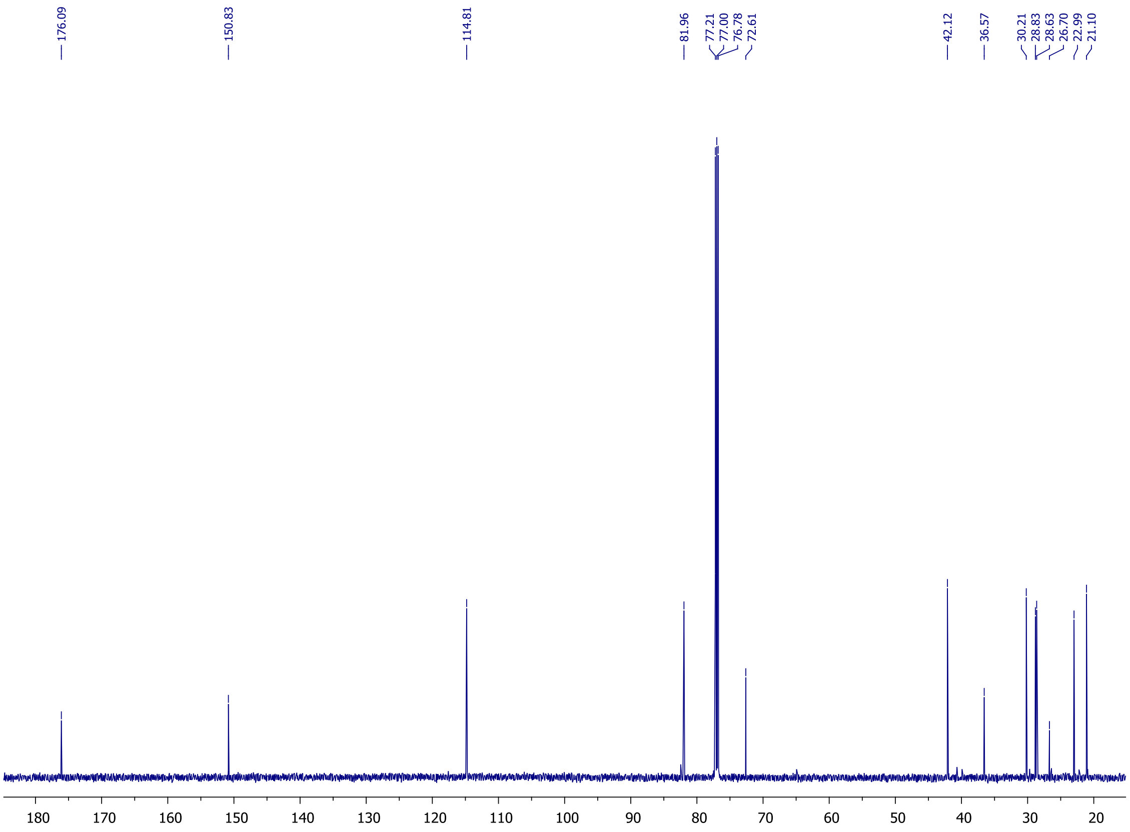

Figure 7. CGC chromatograms of unsaturated lactones: (**a**) (±)-2; (**b**) (+)-2, *ee* = 96%; (**c**) (−)-2, *ee* = 98%.

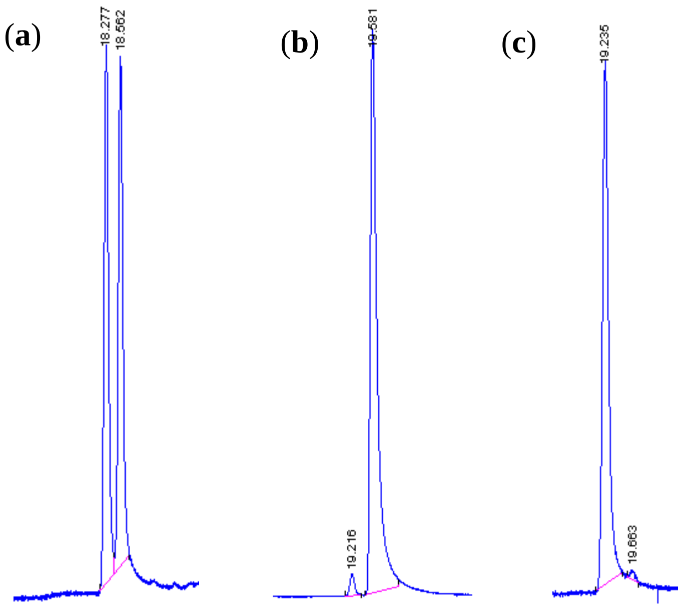

Supplement: Supplementary file 1 [file molecules-18-02778-s001.pdf]
